# *Staphylococcus aureus* ST398 gene expression profiling during *ex vivo* colonization of porcine nasal epithelium

**DOI:** 10.1186/1471-2164-15-915

**Published:** 2014-10-20

**Authors:** Pawel Tulinski, Birgitta Duim, Floyd R Wittink, Martijs J Jonker, Timo M Breit, Jos P van Putten, Jaap A Wagenaar, Ad C Fluit

**Affiliations:** Department of Infectious Diseases and Immunology, Faculty of Veterinary Medicine, Utrecht University, Utrecht, The Netherlands; MicroArray Department, University of Amsterdam, Amsterdam, The Netherlands; Central Veterinary Institute of Wageningen UR, Lelystad, The Netherlands; Department of Medical Microbiology, University Medical Center Utrecht, Utrecht, The Netherlands; Department of Bionanoscience, Delft University of Technology, Delft, The Netherlands; Department of Technology, Leiden University of Applied Sciences, Leiden, The Netherlands; Department of Infectious Diseases and Immunology, Veterinary Faculty, Utrecht University, Yalelaan 1, PO BOX 80165, 3508 TD Utrecht, The Netherlands

**Keywords:** MRSA ST398, Microarray, Colonization, *Ex vivo* model

## Abstract

**Background:**

*Staphylococcus aureus* is a common human and animal opportunistic pathogen. In humans nasal carriage of *S. aureus* is a risk factor for various infections. Methicillin-resistant *S. aureus* ST398 is highly prevalent in pigs in Europe and North America. The mechanism of successful pig colonization by MRSA ST398 is poorly understood. Previously, we developed a nasal colonization model of porcine nasal mucosa explants to identify molecular traits involved in nasal MRSA colonization of pigs.

**Results:**

We report the analysis of changes in the transcription of MRSA ST398 strain S0462 during colonization on the explant epithelium. Major regulated genes were encoding metabolic processes and regulation of these genes may represent metabolic adaptation to nasal mucosa explants. Colonization was not accompanied by significant changes in transcripts of the main virulence associated genes or known human colonization factors. Here, we documented regulation of two genes which have potential influence on *S. aureus* colonization; cysteine extracellular proteinase (*scpA*) and von Willebrand factor-binding protein (vWbp, encoded on SaPIbov5). Colonization with isogenic-deletion strains (Δ*vwbp* and *ΔscpA*) did not alter the *ex vivo* nasal *S. aureus* colonization compared to wild type.

**Conclusions:**

Our results suggest that nasal colonization with MRSA ST398 is a complex event that is accompanied with changes in bacterial gene expression regulation and metabolic adaptation.

**Electronic supplementary material:**

The online version of this article (doi:10.1186/1471-2164-15-915) contains supplementary material, which is available to authorized users.

## Background

*Staphylococcus aureus* is an opportunistic pathogen colonizing the upper respiratory tract and skin of humans and other mammalian species. The nose is considered to be the primary ecological niche of *S. aureus* in humans [1]. Nasal carriage of *S. aureus* has been identified as a risk factor for the development of various infections in humans [1].

In 2004 a new distinct sequence type (ST398) of methicillin-resistant *S. aureus* (MRSA) has been isolated from pigs in the Netherlands [2]. Since then, MRSA ST398 has been detected in pigs, veal calves and poultry around the world [3–5]. The transmission of MRSA ST398 from livestock to humans has been reported in many countries [6, 7] and contact with livestock is recognized as a risk factor for human colonization [4, 8]. Additionally, ST398 isolates may cause infections in humans [9]. However, the mechanisms underlying successful colonization of pigs and other livestock are incompletely understood.

The molecular mechanisms involved in *S. aureus* colonization have been mainly studied using human cell cultures [10] as well as rodent models [11, 12]. *S. aureus* colonization involves many factors [13]. A crucial step of colonization is attachment to eukaryotic cells which involves several essential factors such as: clumping factor B (ClfB), iron-regulated surface determinant protein A (IsdA) and wall teichoic acid (WTA) [13]. Mutants deficient in one of the genes responsible for expressing these components displayed reduced cell-attachment properties *in vitro* [10] and showed reduced colonization in animal models [14–16]. Additionally, a ClfB mutant showed weakened colonization in the nares of human volunteers when compared to wild type bacteria, suggesting that ClfB is one of the main *S. aureus* factors involved in human colonization [17]. In addition to these factors, several other proteins (e.g., SdrC, SdrD, SasG, and FnbpA) appear to be involved in colonization by binding to desquamated nasal epithelial cells which confirms the multi-factorial nature of *S. aureus*–host interactions [18, 19].

The natural occurrence of *S. aureus* in pigs may involve similar colonization factors as assumed for humans. On the other hand, it has been shown that the MRSA ST398 prevalence in pigs is high and that pigs are very rarely colonized by other *S. aureus* lineages. These observations suggest that additional factors may be involved in successful host adaptation and maintenance of colonization of *S. aureus* ST398 in livestock. A study of Viana *et al.* suggested that the presence of an additional von Willebrand binding factor protein (vWbp), encoded on a pathogenicity island (SaPI), represents a host adaptation factor of *S. aureus* for animals [20]. Moreover, it has been suggested that mobile genetic elements (MGEs) play a central role in the adaptation of bacteria to different host species [21].

To study *S. aureus* colonization in pigs, we have developed an *ex vivo* porcine nasal mucosa explants model [22]. Our aim was to identify bacterial factors involved in maintenance of *S. aureus* colonization in pigs by analyzing and documenting global gene expression changes in *S. aureus* during *ex vivo* colonization. This is the first study to examine changes in the complete *S. aureus* transcriptome during experimental colonization which mimics natural MRSA ST398 colonization in pigs.

## Results

### Persistence of MRSA ST398 S0462 on porcine mucosa explants

**Figure 1:**
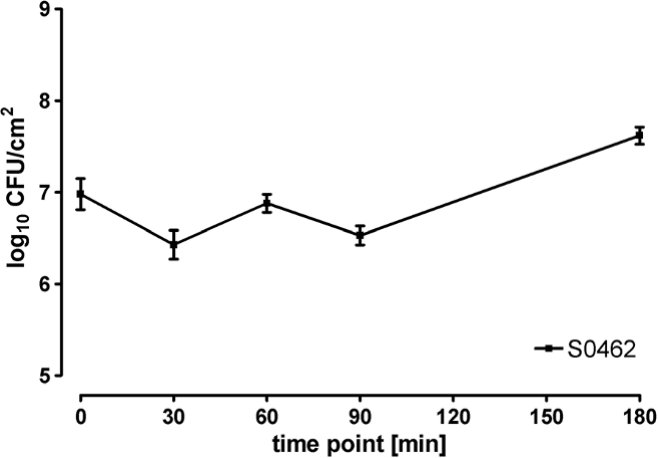
**MRSA S0462 colonization of porcine mucosa explants.** Presence of MRSA S0462 on porcine nasal mucosa explants expressed as CFU on a log scale. Data are presented as the mean CFU ± standard deviation (error bars) of five different experiments with tissue from different pigs.

The ability of *S. aureus* to colonize porcine mucosa explants was defined as persistence or outgrowth of MRSA S0462 on the explants. The explants (1 cm^2^) were inoculated with 1 ml 3 × 10^8^ CFU/ml. After 2 h of incubation (37°C) and washing of the explants, approximately 8 × 10^6^ CFU/cm^2^ (3%) adhered to the explants. The presence of *S. aureus* S0462 on the mucosa explants was followed for an additional 180 min. During the first 30 min of this latter period, MRSA S0462 showed an initial decline in the number of CFU to approximately 3 × 10^6^. Bacterial presence remained stable until 90 min into the experiment. In the following 90 min a significant increase of tissue-associated bacteria to approximately 4 × 10^7^ CFU/cm^2^ was observed (Figure 1). This indicates that MRSA S0462 is able to establish and maintain colonization on the nasal explants during the experiment.

### *S. aureus*transcriptome dynamics during *ex vivo*colonization

To identify genes that possibly contributed to the successful maintenance of colonization of *S. aureus* ST398 in pigs we analyzed the global changes in MRSA S0462 gene expression during *ex vivo* colonization on porcine nasal mucosa explants compared to time-point 0 (directly after removal of unbound bacteria). From MRSA S0462, recovered at different time-points from the explants, RNA was isolated, converted to cDNA and the cDNA hybridized with the *S. aureus* microarray.

**Figure 2:**
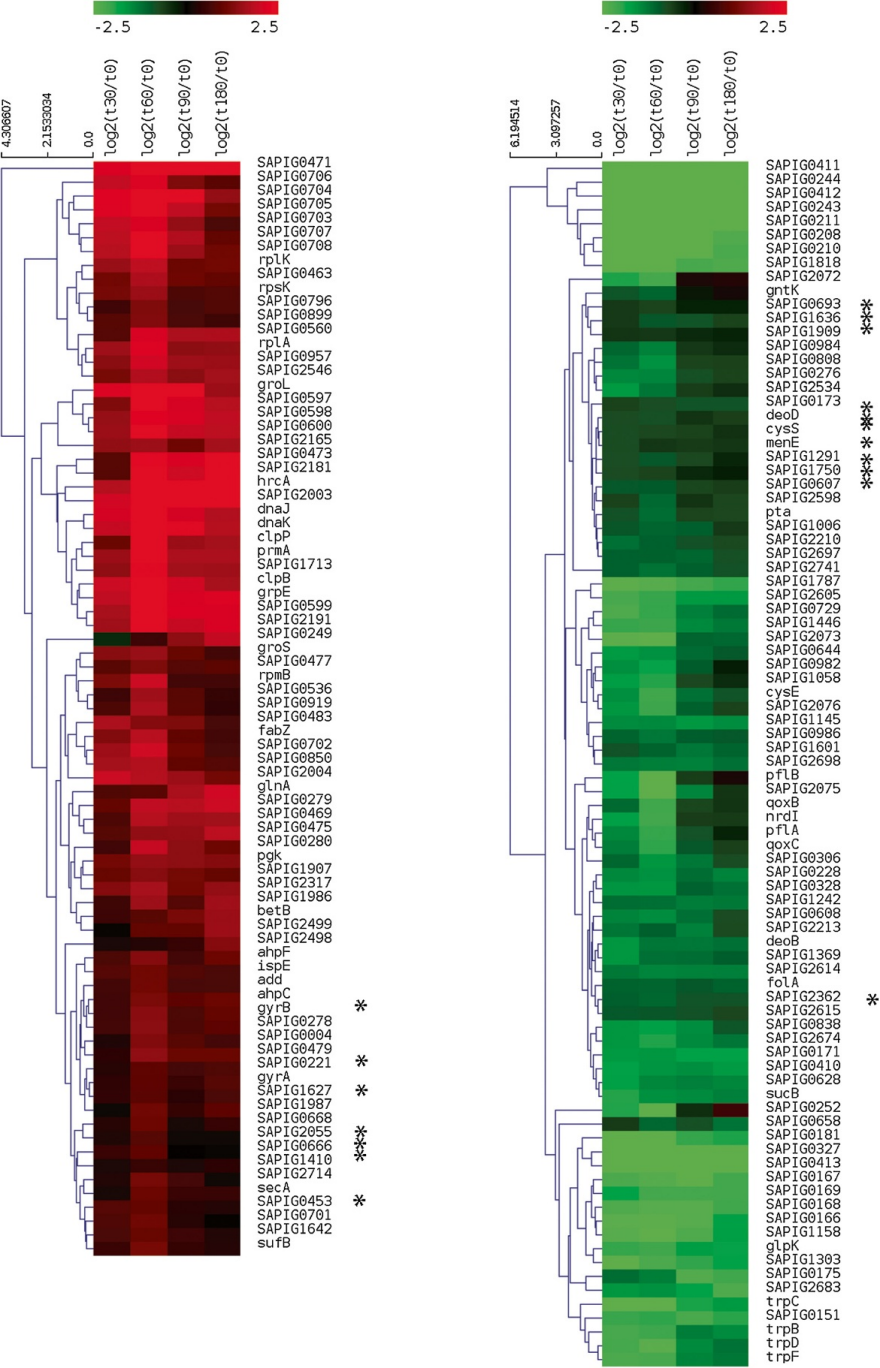
**Heatmap of MRSA S0462 during** ***ex vivo*** **colonization on porcine nasal mucosa explants.** Gene expression profiles of all significantly (p < 0.05) up- and down- regulated gene transcripts in MRSA S0462 during *ex vi*vo colonization. Results are presented as log2 fold-changes compared to time-point t = 0. The columns show the significantly regulated genes, indicated in red (up-regulated) or green (down-regulated) at t = 30 to t = 180.

In total we documented significant changes in the expression of 166 genes compared to time-point 0. Transcripts that were significantly up- or down-regulated (adjusted p = 0.05) were visualized in a hierarchical clustering (Figure 2). Regulation of 148 of these 166 genes was deemed to be biologically relevant (at least 2-fold linear change at least one time-point). A total of 75 genes were negatively regulated and 73 genes were positively regulated (Figure 2 and Additional file 1: Table S2). In general, there were no transcripts that changed in expression direction during the *ex vivo* colonization assay. For the majority of genes the change in expression compared t = 0 min was maximal for t = 60 min, whereas fewer genes showed their maximum expression during t = 30 min and t = 90 min. Only a minority of the genes showed relatively large changes in expression for t = 180 min compared to t = 0 min.

The regulated genes were mapped to available KEGG pathways based on the gene IDs. Most regulated genes were involved in metabolic processes, probably reflecting metabolic adaptation to nasal mucosa explants, of fatty acid biosynthesis, oxidative phosphorylation, and of phenylalanine, tyrosine and tryptophan biosynthesis (Additional file 1: Table S2). In addition, regulation of the expression of genes that are part of the *agr* two-component regulatory system and a number of virulence genes was observed.

The genome of MRSA S0462 contains a vWbp-encoding pathogenicity island, SaPIbov5, which is widely distributed among *S. aureus* isolated from livestock [20]. Only a few genes located on this island were subject to regulation (*int, vwbp* and some of the hypothetical proteins) in our model. It has been suggested that presence of the gene encoding an additional vWbP encoded on the pathogenicity island may play a role in bacterial adaptation to the animal host [20]. During *ex vivo* colonization *vwbp* gene expression was up-regulated to its highest level at t = 60 min (3-fold change). Other genes located on the SaPIbov5 showed a larger change in expression after t = 60 min.

MRSA S0462 *ex vivo* colonization was also accompanied by changes in a number of putative virulence genes. The analysis showed 2.2 to 5.9-fold down-regulation of expression of genes in the *cap* operon encoding capsular biosynthesis during all time-points (2.2 to 5.5-fold change) and of the *hla* gene conferring alpha-hemolysis −3.3 to −5.7-fold change). Up-regulation was observed for the cysteine proteinase cluster (*scpAB*). The expression of the *scpA* gene encoding the cysteine proteinase was up-regulated during the entire experiment (fold change varied from 4.2 to 11.5) and of the *scpB* encoding the cysteine proteinase cellular inhibitor (fold change varied from of 2.2 to 4.0). Moreover, the expression of the genes that constitute the *agr* locus, which encodes a quorum sensing system that controls the expression of virulence genes was also down-regulated mainly at t = 60 min (4.2 to 6.9-fold). However, regulation of *agrD* transcription was not detected.

**Figure 3:**
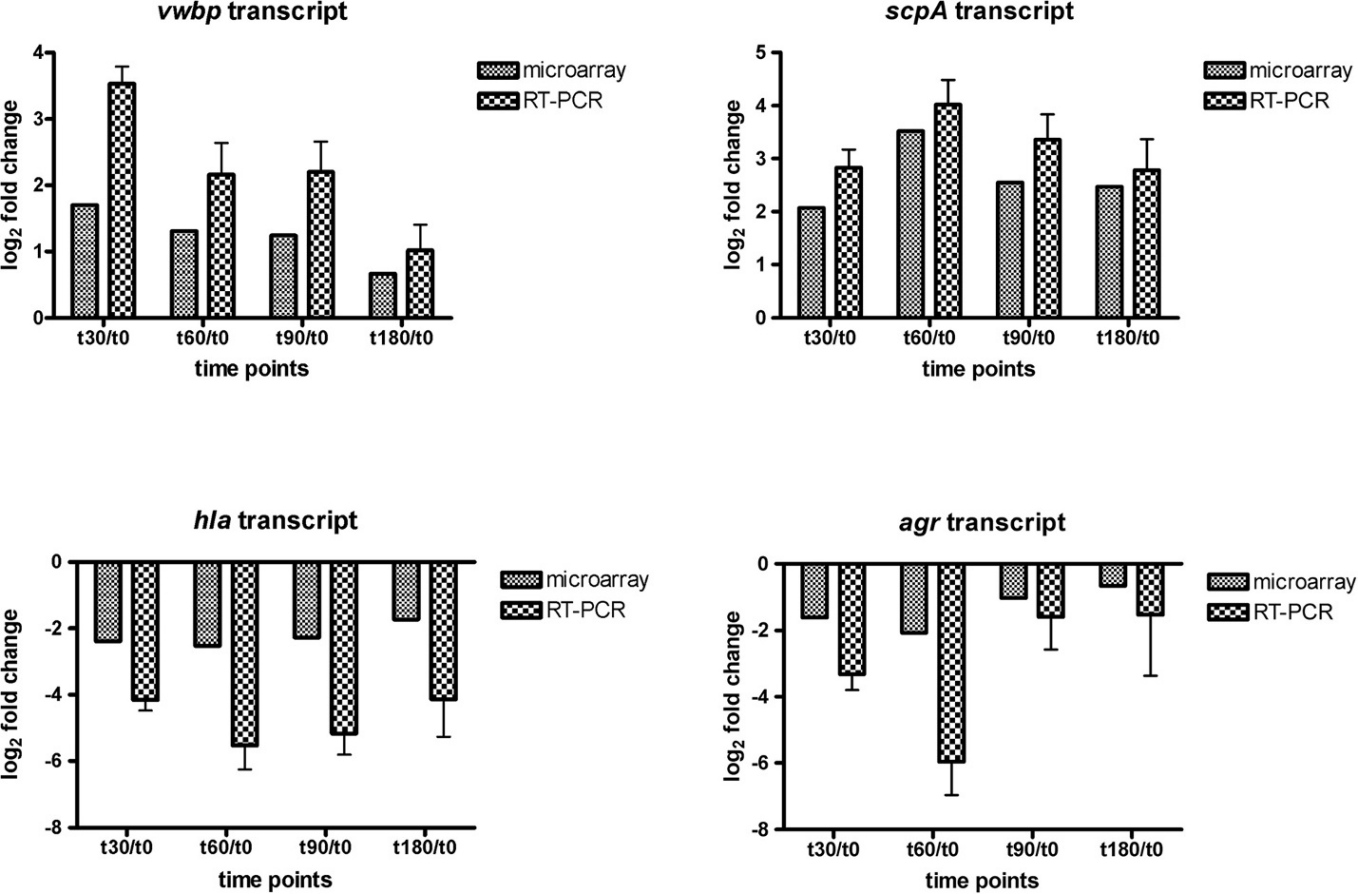
**Changes in expression of four transcripts during** ***ex vivo*** **colonization.** Validation of microarray data by real-time qRT-PCR. Results are expressed as the average log2 fold-change in transcript during *ex vivo* colonization. Data are presented as mean ± standard deviation (error bars) of three independent experiments.

To verify the microarray results, four genes of interest (2 up-regulated genes: *vwbp*, *scpA* and 2 down-regulated genes *agrA*, and *hla*) were subjected to qRT-PCR (Figure 3) using the same RNA samples. The qRT-PCR results confirmed the microarray data (*vwbp* r =0.98, *scpA* r =0.93, *agrA* r =0.95, and *hla* r =0.63), although the fold change values determined by qRT-PCR were higher compared to the microarray data.

*S. aureus ex vivo* colonization did not show regulation of genes encoding surface proteins responsible for attachment of bacteria to the epithelium (*clfB*, *isdA*, and *fnbA)*. To determine whether these genes were expressed during *ex vivo* colonization qRT-PCR analysis was performed. The results showed that the three genes important for human colonization: *clfB*, *isdA*, and *fnbA* were expressed during the colonization of the pig tissue (Additional file 2: Figure S1).

### Contribution of the *vwbp*and *scpA*to *ex vivo*colonization

Next, we generated isogenic *vwbp* and *scpA* deletion mutants of MRSA S0462 (Δ*vwbp* and *ΔscpA*) to investigate the potential role of these genes in MRSA S0462 colonization. The *vwbp* and *scpA* mutants were tested in the *ex vivo* colonization assay (Additional file 3: Figure S 2A and B). Surprisingly, the colonization pattern of the wild-type, Δ*vwbp* and *ΔscpA* mutants did not show significant differences (Additional file 3: Figure S2C). These findings indicate that investigated genes are not crucial for colonization *ex vivo*, but may reflect adaptive changes during the colonization event.

## Discussion

The nostrils are the primary reservoir of *S. aureus* both in humans and pigs. Nasal carriage of *S. aureus* has been identified as a risk factor for the development of various infections in humans [1]. During the last decade, swine have appeared as the major reservoir of MRSA *S. aureus* ST398 which also emerged in other livestock animals. Currently, contact with livestock is recognized as the main risk factor for MRSA ST398 colonization in humans. However, the factors which play a role in the colonization and maintenance of MRSA ST398 in livestock are unclear. *S. aureus* colonization in humans and rodent animal models is a multifactorial process which includes steps like bacterial attachment to the cells, immune escape as well as competition between *S. aureus* and natural flora. Identification of essential factors involved in *S. aureus* maintenance of colonization and adaptation in livestock are difficult to perform in an *in vivo* setting. Previously, we reported the successful establishment of an *ex vivo* model to study MRSA colonization [22]. Using this model we were able to mimic the natural situation in pig’s noses under controlled conditions. During the *ex vivo* colonization, an initial decline in the number of CFU of *S. aureus* S0462 during the first 30 min after inoculation was observed, which indicated that some bacteria still detach from the mucosa or die. Later, a significant increase in the number of bacteria was observed, which indicated maintenance of colonization and even outgrowth of the bacteria. From these data we concluded that establishment of *S. aureus* in our model mimics the initial phases of nasal colonization defined as maintenance and/or outgrowth.

Data on gene expression of *S. aureus* during colonization is limited to the direct transcript analysis of a few genes [23, 24]. Global changes in gene expression during colonization have not been studied before. The major aim of our study was to identify bacterial factors involved in maintenance of colonization by determining and documenting the changes in *S. aureus* gene expression during *ex vivo* colonization.

The expression of 148 biologically relevant genes was regulated during culture on mucosa, when compared to t = 0, the moment that unbound bacteria were removed from the explants. The expression of the majority of genes was highly regulated at 60 min after inoculation. At this time-point a net loss of bacteria is changing to a net increase mimicking colonization.

Based on at least a two-fold change in expression, MRSA colonization apparently resulted in changes in regulation of metabolic pathways. The biosynthesis pathways for phenylalanine, tyrosine and tryptophan biosynthesis pathways were down-regulated, in contrast to the genes involved in the main amino acid biosynthesis pathways which were not regulated in our system. The active protein biosynthesis, together with lack of regulation of the *de novo* amino acid biosynthesis pathways indicates that the bacterial cells have access to these amino acids. The *ex vivo* colonization was performed at an air-liquid interface, where bacteria may have had contact with the cultivation medium (RPMI and DMEM) containing free amino acids which may have served as a source of amino acids for bacteria, although we carefully avoided the presence of culture medium on top of the explants.

A crucial step of *S. aureus* nasal colonization is adhesion to epithelial cells. It has been shown that ClfB, IsdA proteins and WTA are essential factors for *S. aureus* adherence to human cell lines *in vitro* [10, 19], nasal colonization of rodents [14, 16, 25], and for nasal colonization of humans [17]. After initial adherence, during the first 30 min MRSA S0462 showed an initial decline in the number of CFU and significant increase of tissue-associated bacteria in later phase of the experiment consistent with colonization. In the pig nasal *ex vivo* system we did not observe regulation of expression of the adhesion genes, however the genes were expressed. In our settings, bacteria used for tissue inoculation were harvested in the mid-log phase, where adhesion factors are well expressed. Therefore, contact with the nasal epithelium did not result in regulation of the expression of genes encoding adherence factors such as *clfB*, *isdA*, and *fnbA*. The alternative explanation, that the relevant mRNAs were not detected is unlikely based on the results of the data analysis and experience with highly similar microarray experiments for *S. aureus* [26].

Expression of the most adhesins is under the regulation of the *agr* system [23]. The *agr* system has a dual action in the global gene regulation in *S. aureus*. This system positively regulates toxins, extracellular proteases, immunomodulation factors and capsule biosynthesis, but represses the expression of some of the surface proteins such as protein A, coagulase and fibrinogen binding protein past the exponential phase. During *ex vivo* colonization the *agr* locus was down-regulated. However, the *agrD* gene, part of the *agr* locus, was not significantly regulated. This gene may be expressed at a basal level without a change in regulation. Additionally, we observed down-regulation of *hla* and the *cap* operon, which are controlled by the *agr* system (down-regulated). Burian *et al.* had similar findings showing that during *S. aureus* colonization of the human nose and in cotton rats the *agr* system is weakly expressed [23, 24].

Interestingly, the cysteine proteinase operon (*scpAB*) was up-regulated during the whole experiment. ScpA is known to cleave a number of extracellular matrix components and it has been suggested to play a role in bacterial migration from the sites of initial colonization [27, 28], but it may also play a role in the acquisition of nutrients [29]. It has been suggested that the extracellular proteases can promote *S. aureus* skin and nares colonization by degradation of some *S. aureus* virulence factors like toxins [30]. Moreover, it has been shown that *S. aureus* ScpA protease is associated with diseases such as Staphylococcal Scalded Skin Syndrome [31] and is involved in vascular leakage causing sepsis [27]. The up-regulation of the extracellular proteinase ScpA indicates that this protein may be involved in establishment of colonization *ex vivo*. To study the influence of the cysteine proteinase for colonization *ex vivo*, we generated a ScpA isogenic mutant. However, a single knockout mutant did not show any phenotypic difference in colonization pattern *ex vivo*, which indicates that ScpA does not play a crucial role in colonization/adaptation *ex vivo*.

Also the up-regulation of some genes located on SaPIbov5 was observed. This mobile element encodes an additional von Willebrand factor binding protein. It has been shown that *S. aureus* harboring a pathogenicity island with the additional *vwbp* are widely distributed in ruminants and it has been suggested that this protein is one of the adaptation factors for *S. aureus* to animal hosts [20]. Our study showed that during *S. aureus* ST398 interaction with porcine nasal epithelium the *vwbp* was strongly up-regulated during the first phase of the experiment (t = 30 min), where the initial decline of CFU was observed. This may indicate that *vwbp* may be important in *S. aureus* adaptation to the porcine nasal epithelium and may promote *S. aureus* colonization *ex vivo*. However, the single knockout mutant did not show any phenotypic difference in colonization pattern *ex vivo*, which indicates that *vwbp* also does not play a crucial role in colonization/adaptation *ex vivo*.

In this study, we successful applied an *ex vivo* model to study changes in the gene expression regulation during colonization using one reference ST398 strain. Our previous study showed in an *ex vivo* model, colonization of different MRSA types from animal and human origin [22]. A study of Szabo *et al.* also reported a slight difference in pig colonization between different MRSA types [32]. Further study on gene expression of different *S. aureus* strains, during *ex vivo* colonization should be performed to understand strain specific colonization mechanisms.

There are few limitations of our model, which may influence *S. aureus* gene expression. First of all, adhesion of the bacteria onto the tissue was performed in DPBS. Furthermore, the host immune response will be absent, which might manifest itself in lack of regulation of the immune escape genes and influence *S. aureus* gene expression. Moreover, in our system we are not able to preserve the natural microbiota present in nasal mucosa tissue. In natural conditions, *S. aureus* must also compete with other bacterial species, which also would influence the global changes in gene expression. Recently, it has been reported that in humans, these interactions in the microbiota of the nasal cavity, influence the colonization of *S. aureus* [33]. Investigation on the microbiota in the nasal cavity of pigs and the bacteria interaction is necessary to understand the colonization process of *S. aureus*.

## Conclusions

We could for the first time establish changes in the global gene expression pattern of *S. aureus* during *ex vivo* colonization. Additionally, this study suggests that nasal colonization with MRSA ST398 is a complex event that is accompanied with changes in bacterial gene expression regulation and metabolic adaptation.

## Methods

### Ethics statement

Tissue was isolated from pigs that were euthanized after a cardiovascular study at the UMCU (Utrecht, the Netherlands), which was approved by the Utrecht Animal Ethics Committee in accordance with article 18 of the Dutch Experiments on Animals Act and carried out in accordance with the European Guidelines for the accommodation and care of animals used for experimental and other scientific purposes as laid down in EU Recommendation 2007/526/EC.

### Bacterial strain and *ex vivo*colonization assay

MRSA ST398 S0462 strain (*spa*-type: t011, SCC*mec* IV) was taken for the transcription experiments because in a previous study it was shown to adhere to and grow on pig nasal explants. S0462 was isolated from a colonized pig. The strain was sequenced and the sequence was submitted to EMBL (accession numbers CAVX010000001-CAVX010000134) [34]. The preparation of nasal mucosa explants from pigs was performed as previously described [22]. The explants were inoculated with MRSA isolates as described previously [22]. Briefly, *S. aureus* strains were grown overnight in BHI at 37°C. A 2% aliquot was inoculated into fresh 10 ml broth and grown at 37°C under shaking (200 rpm) to mid-exponential phase (approximately 4 h). Bacteria were harvested by centrifugation at 3,750 × *g* for 5 min, washed 3 times in Dulbecco’s Phosphate-Buffered Saline (DPBS), and suspended at an OD_600_ of 0.6 (approximately 3 × 10^8^ CFU/ml) in DPBS. The explants were inoculated with 1 ml of bacterial suspension in DPBS (approximately 3 × 10^8^ colony forming units (CFU)/ml in DPBS) for 2 h to allow the bacteria to adhere to the tissue (adhering time). Next, bacteria were washed with DPBS to remove unbound bacteria and explants were cultivated at 37°C in a 5% CO_2_ atmosphere. At different time-points (0, 30, 60, 90, and 180 min) after removal of unbound bacteria, samples for RNA extraction and subsequent transcription analysis were taken. At these time-points explants were washed three times with 1 ml DPBS. Bacteria were isolated from the explants by scraping the epithelium surface using cell scrapers (Falcon, Becton Dickinson, The Netherlands) and resuspended in 1 ml of DPBS with 0.1% Triton X-100. Nine hundred μl of the bacterial suspension was immediately centrifuged at 20,000 × *g* for 2 min (room temperature) and the resulting pellet was frozen at −80°C before RNA isolation. The remaining 100 μl of bacterial suspension was serially diluted in DPBS and plated on blood agar plates (Oxoid, UK). The plates were incubated overnight at 37°C and CFU were enumerated after 24 h. The colonization assay was repeated independently four times. Additionally, to investigate MRSA adaptation to mucosa, the suspension was incubated using the same growth conditions, but without contact with an explant. After 2 h bacteria were collected for RNA isolation.

### RNA extraction

RNA was purified using the NucleoSpin RNA II total RNA isolation kit (Macherey-Nagel, Germany) according to manufacturer’s protocol with some adjustments as described [26]. No removal of eukaryotic RNA was performed as the specificity of the microarray was high enough to discriminate between different *S. aureus* strains.

### Microarray design

The microarray was specifically developed for multiple *S. aureus* strains. The complete design was performed in a two-step procedure. First, 60-mer oligonucleotides were designed each 40 base pairs on alternating strands for the first sequence, ST398. Second, for each next sequence oligonucleotides were only designed for regions that were not probed by previously designed oligonucleotides. Oligonucleotides that match a sequence with a bitscore over 80 were considered as usable for probing. Using these parameters 121,901 probes were generated and manufactured as a microarray by Nimblegen (Roche) in a 12 × 135 K format.

For more details see the Additional file 4.

### Labeling of total RNA, hybridization and scanning

Total RNA was labeled with fluorescent dyes by an amplification procedure and direct labeling. A total of 100 ng RNA was used as input for the Ovation Pico WTA System according to manufacturer’s instructions (Nugen Technologies, Inc, USA). Two μg of purified and amplified cDNA was used as input for labeling by randomly priming with Superscript II reverse transcriptase (Invitrogen, The Netherlands), random octamers (100 ng/μl) and actinomycine D, in a total volume of 10 μl, for 2 h at 42°C with the incorporation of Cy5- or Cy3-dUTP (Amersham, USA) with a ratio dUTP/dTTP of 3/1. Labeled cDNA was purified using Qia-quick PCR purification kit (Qiagen, USA). Incorporation of Cy3 or Cy5 was determined using a NanoDrop ND-1000.

The common reference was created by pooling Cy5-labeled RNA samples. Labeled cDNA was hybridized according to manufacturer’s protocol (Roche NimbleGen, The Netherlands). A total of 1.1 μg Cy3-labeled cDNA and 1.1 μg Cy5-labeled common reference was mixed in 7.2 μl of NimbleGen hybridization cocktail. The mixture was heated to 65°C for 5 min, and a total of 6 μl was loaded onto the custom made *S. aureus* array and hybridized for 18 h at 42°C in a dedicated hybridization chamber (Roche NimbleGen, The Netherlands).

After the hybridization the arrays were dismantled at 42°C and washed in buffer 1 for 2 min at room temperature, then 1 min in wash buffer 2 at room temperature and finally 15 sec in wash buffer 3 at room temperature (Roche NimbleGen, The Netherlands). Slides were spun for 30 sec at 300 rpm to dry and scanned immediately in an Agilent DNA MicroArray Scanner. Data was extracted and processed using NimbleScantm software (version 2.6, Roche NimbleGen, The Netherlands).

### Data analysis and statistical analyses

Processing of the data was performed using R (version 2.14.1) and the Bioconductor MAANOVA package (version 1.10.0). All slides were subjected to a set of quality control checks, which consisted of visual inspection of the scans, examination of the consistency among the replicated samples by principal component analysis (PCA), testing against criteria for signal to noise ratios, testing for consistent performance of the labeling dyes and visual inspection of pre- and post-normalized data with box and ratio-intensity plots. After log2 transformation, the data were normalized by a within-slide LOWESS smoothing procedure. The features were annotated for *S. aureus* ST398 using GenBank, and gene expression values were calculated using the robust multi-array average (RMA) algorithm [35], which also performs between slide normalization. The resulting data were analyzed using an ANOVA model. A contrast analysis was applied to compare the samples from each time point with the control group (t = 0) [36]. For hypothesis testing a permutation based Fs test was used [37] and the resulting P-values were corrected for false discoveries according to Storey and Tibshirani [38]. The significance threshold was set at 0.05 FDR. A complete set of the microarray data has been deposited at the GEO database (accession number GSE47910). Changes in expression of at least 2-fold linear change were arbitrarily considered biologically relevant.

For more details see the Additional file 4 and Jonker *et al*. [39].

### Real-time quantitative reverse transcriptase PCR

RNA samples used in the microarray experiments were also analyzed by quantitative reverse-transcriptase real-time PCR (qRT-PCR). Purified *S. aureus* RNA was converted using the Bacterial H-TR cDNA synthesis kit (AmpTech, Germany). The cDNA products were subsequently used in qRT-PCR using SYBR Green Master Mix for qRT-PCR (Takara Bio Inc, Japan) according to the manufacturer’s instruction and the qRT-PCR reaction was performed in a LightCycler 480 (Roche, The Netherlands). The transcripts for *vwbp*, *scpA*, *hly* and *agr* were amplified using primers listed in Additional file 5: Table S1. All signals were normalized to *aroE* and *gmk* gene transcripts (both housekeeping genes) [40]. Relative quantification of gene expression was calculated using the comparative cycle threshold method as described previously by Livak and Schmittgen [41]. Data obtained are expressed as the mean log_2_ fold-change in transcript during colonization *ex vivo* for selected post time-points after removal of unbound bacteria (30, 60, 90, and 180 min) relative to the t =0 control sample. All time-points were tested in triplicate.

### Construction of an isogenic *vwbp*and *scpA*deletion strain (Δ*vwbp*and Δ*scpA*)

Isogenic *vwbp* and *scpA* deletions in the S0462 strain were generated by allelic replacement as described [42] with a slight modification. Briefly, regions around 1000 bp flanking the *vwbp* and *scpA* locus were amplified by PCR using primers listed in Additional file 5: Table S1. The resulting PCR fragments were used as a template to create an insertion fragment by PCR overlap. Next, the PCR fragment was digested using *Eco*RI and *Not*I restriction endonucleases (Fermentas, Lithuania) and cloned into pKOR1 vector. The ligation product was transformed into competent *Escherichia coli* DC10b cells [43] and grown on LB agar containing 100 μg/ml ampicillin. Purified plasmid (500 ng) containing the correct insert (confirmed by sequencing) was electroporated into the target strain, *S. aureus* S0462, using the settings: 200 Ω, 25 μF and 1.5 kV. After electroshock 200 μl of TSB was added and cells were incubated at 37°C for 1 h with shaking, to allow recovery. The cells were plated on TSA plates containing 7.5 μg/ml chloramphenicol and grown overnight at 30°C. Single colonies were grown in TSB overnight at 30°C with vigorous shaking. Plasmid integration was checked by overnight culture at 43°C on TSA with 7.5 μg/ml chloramphenicol. Colonies were screened for single cross-over by PCR. Single cross-over mutants were grown in TSB without antibiotic at 30°C overnight, diluted 1:100,000 in sterile water and 100 μl was spread on TSA plate containing 50 μg/ml anhydrotetracycline and incubated at 37°C overnight. Large colonies were picked and cultured overnight at 37°C on TSA with 10 μg/ml chloramphenicol and plain TSA. Colonies growing only on plain TSA were assumed to be knock-out mutants. Putative mutants were validated by PCR amplification and sequencing of genomic DNA flanking the deletion. Confirmed knock-out strains were used in subsequent experiments.

### Availability of supporting data

The microarray data has been deposited at the GEO database (accession number GSE47910). http://www.ncbi.nlm.nih.gov/geo/query/acc.cgi?token=xzojxwqwuiaqqru&acc=GSE47910.

## Electronic supplementary material

Additional file 1: Table S2: Changes in the MRSA S0462 transcriptome during *ex vivo* colonization. Microarray results are presented as the mean linear fold-change from six separate experiments. Only transcripts with significant fold-change (*p* value < 0.05 -fold change in transcriptome) are included. The results are expressed as the average linear fold-change in transcript during *ex vivo* colonization compared to t = 0. (XLS 30 KB)

Additional file 2: Figure S1: Expression of three important colonization genes: *clfB, isdA*, and *fnbA* during *ex vivo* colonization. The qRT-PCR results are expressed as the average log2 fold-change in transcript during *ex vivo* colonization compared to t = 0. (TIFF 954 KB)

Additional file 3: Figure S2: Construction and characterization of a knockout *vwb* and *scpA* strains in MRSA S0462. A) Schematic representation of the deletion *vwb* or *scpA* genes in MRSA S0462 strain. B) PCR confirmation of *vwb* and *scpA* deletion mutagenesis. C) MRSA S0462 wild-type, Δ*vwb*, and Δ*scpA* colonization of porcine mucosa explants. Data are presented is the mean log CFU ± standard deviation (error bars) of three different pig experiments. (TIFF 2 MB)

Additional file 4: **Supporting information.** (DOC 204 KB)

Additional file 5: Table S1: Primers, plasmids and strains used in this study. (DOC 69 KB)
