# Supplementary figures and images for: Staphylococcus aureus ST398 gene expression profiling during ex vivo colonization of porcine nasal epithelium
Source: BMC Genomics. 2014 Oct 20;15(1):915. doi: 10.1186/1471-2164-15-915 (PMC4210494; doi:10.1186/1471-2164-15-915)

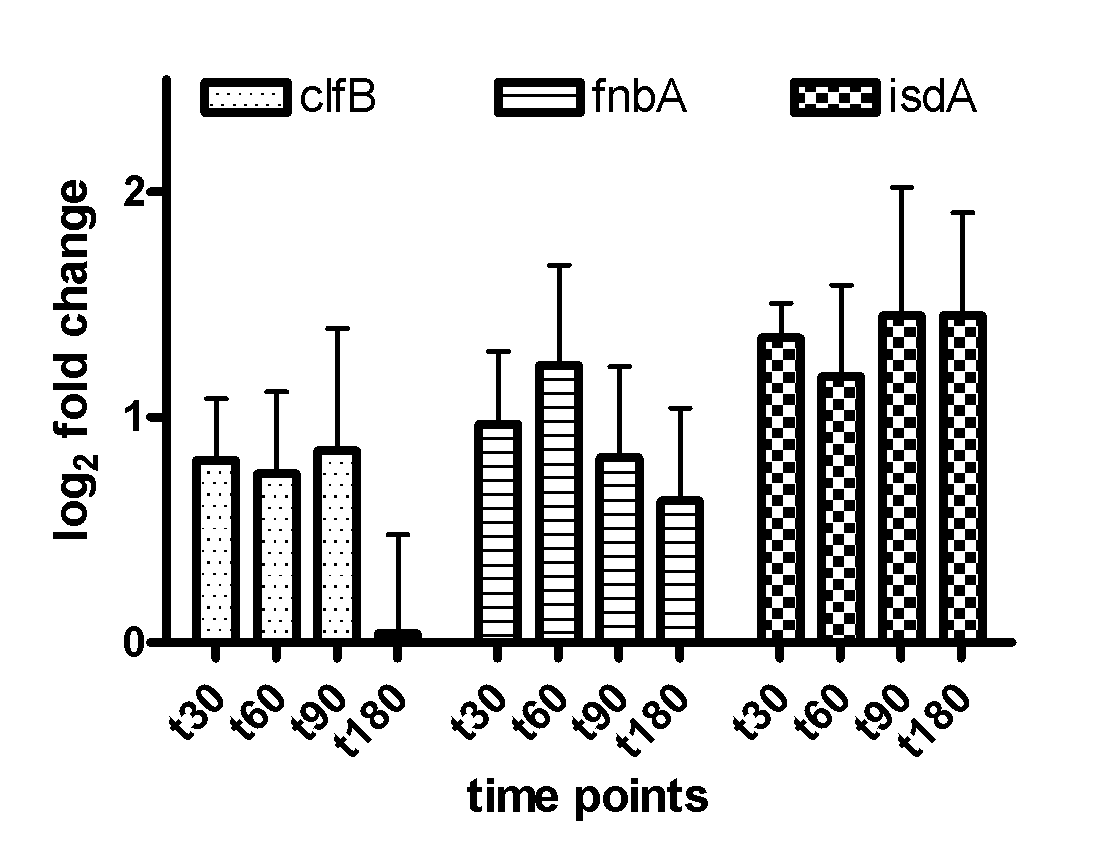

Supplement: Supplementary file 2 — Additional file 2: Figure S1: Expression of three important colonization genes: clfB, isdA, and fnbA during ex vivo colonization. The qRT-PCR results are expressed as the average log2 fold-change in transcript during ex vivo colonization compared to t = 0. (TIFF 954 KB) [file 12864_2014_6602_MOESM2_ESM.tiff]

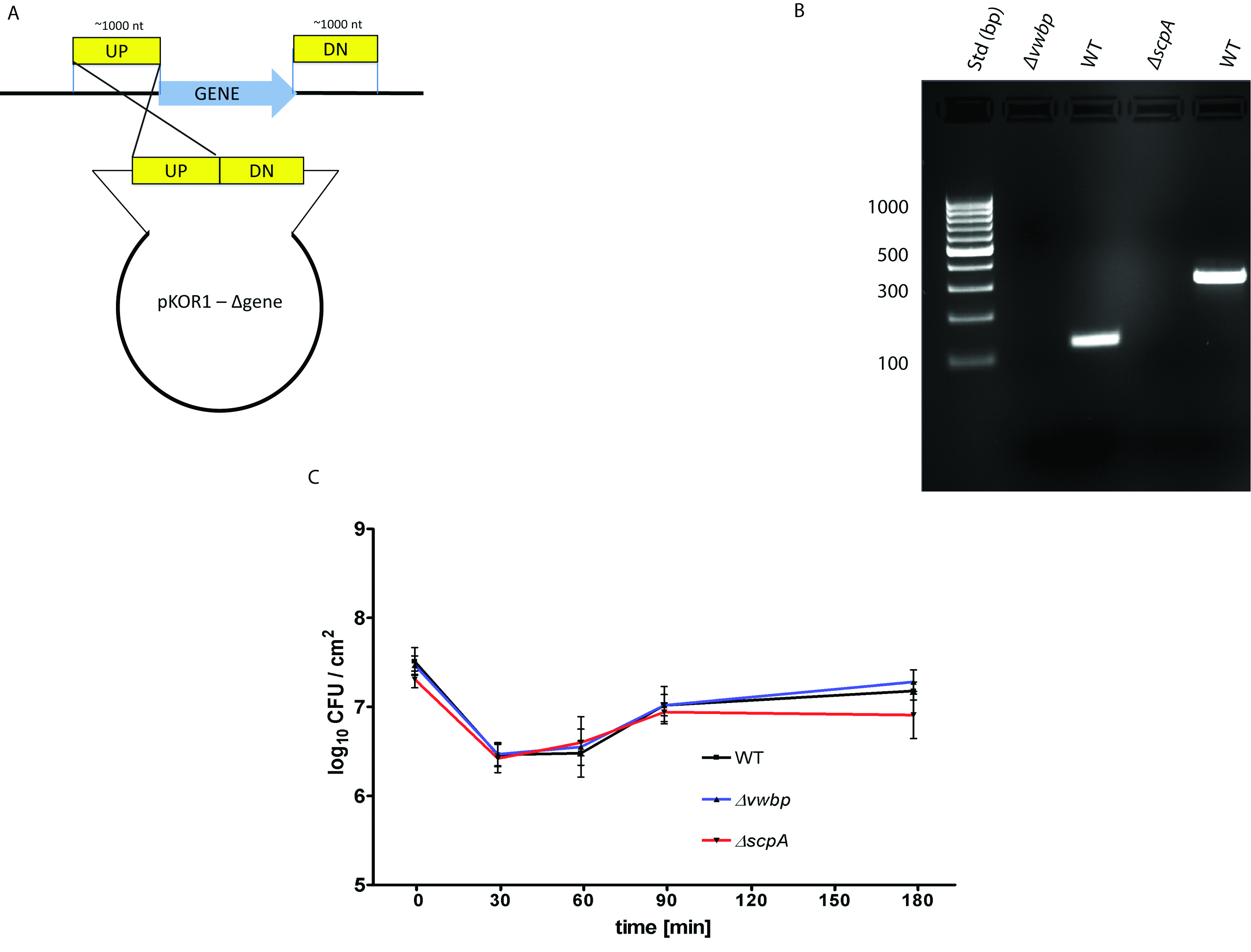

Supplement: Supplementary file 3 — Additional file 3: Figure S2: Construction and characterization of a knockout vwb and scpA strains in MRSA S0462. A) Schematic representation of the deletion vwb or scpA genes in MRSA S0462 strain. B) PCR confirmation of vwb and scpA deletion mutagenesis. C) MRSA S0462 wild-type, Δvwb, and ΔscpA colonization of porcine mucosa explants. Data are presented is the mean log CFU ± standard deviation (error bars) of three different pig experiments. (TIFF 2 MB) [file 12864_2014_6602_MOESM3_ESM.tiff]
