# Supplementary material for: Staphylococcus aureus ST398 gene expression profiling during ex vivo colonization of porcine nasal epithelium
Source: BMC Genomics. 2014 Oct 20;15(1):915. doi: 10.1186/1471-2164-15-915 (PMC4210494; doi:10.1186/1471-2164-15-915)
Supplement: Supplementary file 4 — Additional file 4: Supporting information. (DOC 204 KB) [file 12864_2014_6602_MOESM4_ESM.doc]

**Additional file 4 SUPPORTING INFORMATION**

**MATERIALS & METHODS**

**Micro-array design**

As indicated, the microarray was developed for multiple *S. aureus* strains. The array is based on the sequencing information from 2009, which is the year in which the array was designed. The array contains 121,901 tiling non-replicated probes for the following strains, plasmids and phages: ST398 (Genome), MSSA476 (Genome), MRSA252 (Genome), N315 (Genome), Mu50 (Genome), COL (Genome), MW2 (Genome), RF122 (Genome), USA300 (Genome), NCTC8325 (Genome), JH9 (Genome), JH1 (Genome), Newman (Genome), Mu3 (Genome), TCH1516 (Genome), EMRSA15 (Genome), MN8 (Genome unfinished), TCH60 (Genome unfinished), H1374 (Genome unfinished), JKD6009 (Genome unfinished), NOH4 (Genome unfinished), Sa_0582 (Genome unfinished), SA130/ST-72 (Genome unfinished), TCH70 (Genome unfinished), CF-Marseille (Genome unfinished), Mu50-omega (Genome unfinished), JKD6008 (Genome unfinished), MSSA476 plasmid pSAS (Plasmid), N315 plasmid pN315 (Plasmid), Mu50 plasmid VRSAp (Plasmid), COL plasmid pT181 (Plasmid), plasmid pMW2 (Plasmid), USA300 plasmid pUSA01 (Plasmid), USA300 plasmid pUSA02 (Plasmid), USA300 plasmid pUSA03 (Plasmid), JH9 plasmid pSJH901 (Plasmid), JH1 plasmid pSJH101 (Plasmid), TCH1516 plasmid pUSA300HOUMR (Plasmid), TCH1516 plasmid pUSA01-HOU (Plasmid), USA300_TCH959 plasmid pUSA300HOUMS (Plasmid), plasmid EDINA (Plasmid), EMRSA15 plasmid (Plasmid), plasmid pRJ6 (Plasmid), plasmid pVGA (Plasmid), plasmid pTZ4 (Plasmid), plasmid pSA1379 (Plasmid), phage 37 (Phage), phage phiETA2 (Phage), phage phiETA3 (Phage), phage phiMR11 (Phage), phage phiMR25 (Phage).

The data has been deposited at GEO and has accession number GSE47910. It is accessible with the following link: http://www.ncbi.nlm.nih.gov/geo/query/acc.cgi?token=xzojxwqwuiaqqru&acc=GSE47910

**Data analysis and statistical analyses**

Quality control. The data passed our quality criteria. This is obviously somewhat subjective, but here are some snapshots of pre-normalized data from our QC pipeline:


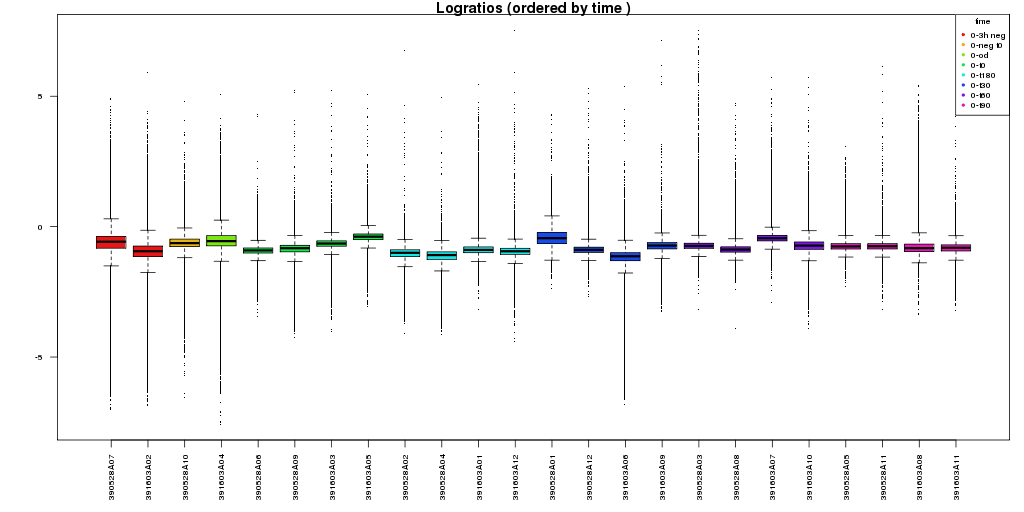

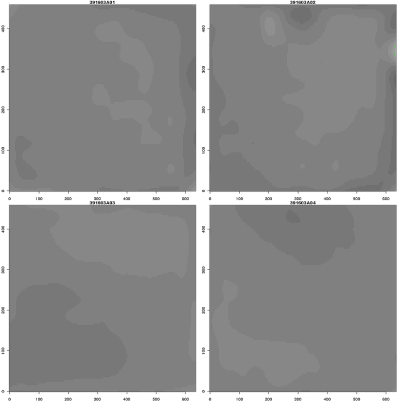


Shown are pseudocolor plots (left) and boxplots for log-ratio’s (right). Some spatial effects were observed (left), but the data looked trustworthy (right).

Details about the data processing. After log2 transformation, the data were normalized by a within-slide LOWESS smoothing procedure. The features were annotated for *Staphylococcus aureus* ST398 and gene expression values were calculated using the robust multi-array average (RMA) algorithm, which also performs between slide normalization. In short, the RMA algorithm performs a background subtraction, a quantile normalization and performs a median polish calculation to collapse the intensities of the probe sets that target one gene into a single value [1].

Statistical evaluation. The gene-specific p-values, which were calculated using an ANOVA procedure, were adjusted to take into account the fact that we are performing 2469 tests (namely one for each gene), rather than a single significance test. The reviewer may be aware of the fact that without such a correction, a 5% significance cut-off will result in ~125 seemingly differentially expressed genes purely by chance alone, also without any gene expression response. As indicated in the manuscript, we used the procedure developed by Storey and Tibshirani [2] and calculated adjusted p-values. The interpretation of these p-values is: the expected proportion of Type I errors among the rejected hypotheses. For instance, if you find 100 differentially expressed genes after performing FDR correction with an α_e_=0.05, then you know that you have five false-positives among these 100 genes.

Proper statistical inference entails evaluating whether genes have high fold-changes relative to the noise, in other words: the ratio fold-change/standard error (FC/SE) has to be high. This is what the t-statistic (and F-statistic) quantifies. A low p-value indicates that the fold-change is high, relative to the (biological and/or technical) noise. As a consequence, low fold-changes are significant if they are measured with little noise. This obviously indicates that the quantification of the fold-change is reproducible.

We used a FDR-corrected p-value of 5% as significance cut-off, and this is relatively stringent. We have chosen to do so, because we wanted to be sure about our conclusions.

The results of a principal components analysis (PCA) look like this:


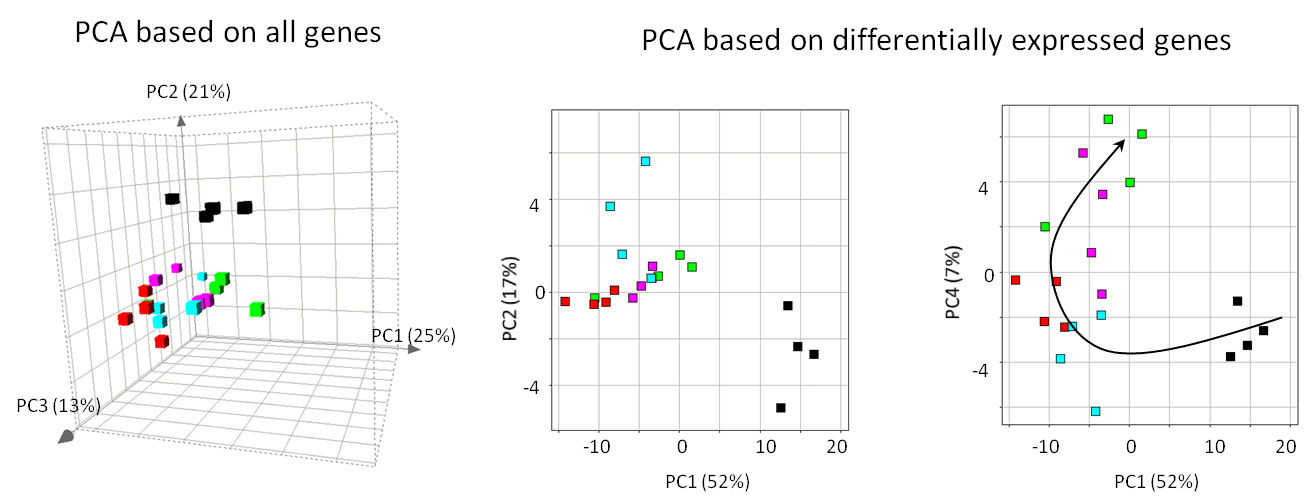


The colors indicate: t=0 (black), t=30 (blue), t=60 (red), t=90 (purple) and t=180 (green).

The time effects are subtle in comparison with the overall variability. The most left figure, based on all genes, shows that we cannot observe a time specific effects in the first three principal components (PC’s): the four time points behave largely like one experimental group. Yet, the control (t=0) samples clearly separate from the other time points. Also when the effect of the differentially expressed genes are examined, we cannot identify a clear time related effect in the first two PC’s (middle picture). However, when the first PC is plotted against the fourth PC, a time effect is observed. The time points are more or less ordered sequentially, as indicated by the arrow in the most right plot. We therefore believe that the differentially expressed genes do play a role during colonization.

References

1. Irizarry RA, Hobbs B, Collin F, Beazer-Barclay YD, Antonellis KJ, Scherf U, Speed TP: **Exploration, normalization, and summaries of high density oligonucleotide array probe level data.** *Biostatistics (Oxford, England)* 2003, **4:**249-264.
2. Storey JD, Tibshirani R: **Statistical significance for genomewide studies.** *Proceedings of the National Academy of Sciences of the United States of America* 2003, **100:**9440-9445.

3. Burian M, Rautenberg M, Kohler T, Fritz M, Krismer B, Unger C, Hoffmann WH, Peschel A, Wolz C, Goerke C: **Temporal expression of adhesion factors and activity of global regulators during establishment of *Staphylococcus aureus* nasal colonization.** *The Journal of infectious diseases* 2010, **201:**1414-1421.

5. Hirschhausen N, Schlesier T, Peters G, Heilmann C: **Characterization of the modular design of the autolysin/adhesin Aaa from *Staphylococcus aureus*.** *PloS one* 2012, **7:**e40353.

6. Bae T, Schneewind O: **Allelic replacement in *Staphylococcus aureus* with inducible counter-selection.** *Plasmid* 2006, **55:**58-63.

7. Monk IR, Shah IM, Xu M, Tan MW, Foster TJ: **Transforming the untransformable: application of direct transformation to manipulate genetically *Staphylococcus aureus* and *Staphylococcus epidermidis*.** *mBio* 2012, **3:**10.1128/mBio.00277-00211. Print 02012.
