# Supplementary material for: Staphylococcus aureus ST398 gene expression profiling during ex vivo colonization of porcine nasal epithelium
Source: BMC Genomics. 2014 Oct 20;15(1):915. doi: 10.1186/1471-2164-15-915 (PMC4210494; doi:10.1186/1471-2164-15-915)
Supplement: Supplementary file 5 — Additional file 5: Table S1: Primers, plasmids and strains used in this study. (DOC 69 KB) [file 12864_2014_6602_MOESM5_ESM.doc]

**Additional file 5: Table S1 Primers, plasmids and strains used in this study.**

| Primer | Sequence / description / characteristic | Function | Reference |
| --- | --- | --- | --- |
| vwb_F | GTGACTGGAGAAGCAAAC | qRT-PCR | This study |
| vwb_R | GCGTGCCTATTTACAGAG | qRT-PCR | This study |
| hla90 | AGAAAATGGCATGCACAAAAA | qRT-PCR | [3] |
| hla488 | TATCAGTTGGGCTCTCTAAAA | qRT-PCR | [3] |
| scpA_ F: | CACCCAAACTTACAAGGACAAC | qRT-PCR | This study |
| scpA_R: | CGCCATTGGATACTGGAATAAC | qRT-PCR | This study |
| agr-1189 | CGATGTTGTTTACGATAGC | qRT-PCR | [4] |
| agr-1436 | CGACACAGTGAACAAATTC | qRT-PCR | [4] |
| gmk_F | AAGGTGCAAAGCAAGTTAGAA | qRT-PCR | [5] |
| gmk_R | CTTTACGCGCTTCGTTAATAC | qRT-PCR | [5] |
| aroE_F | CTATCCACTTGCCATCTTTTAT | qRT-PCR | [5] |
| aroE_R | ATGGCTTTAATATCACAATTC | qRT-PCR | [5] |
| clfB-136 | ATAGGCAATCATCAAGCA | qRT-PCR | [4] |
| clfB-270 | TGTATCATTAGCCGTTGTAT | qRT-PCR | [4] |
| fnbA-160 | GGAGCAGCATCAGTATTCTT | qRT-PCR | [4] |
| fnbA-308 | AGTTGCAGTTGTTTGTGTTT | qRT-PCR | [4] |
| isdA-2665 | GCAGTTACAGCAGGTTTA | qRT-PCR | [4] |
| isdA-2782 | CAGCAAAACCAAACAATG | qRT-PCR | [4] |
| UP-F_vwb_EcoRI | GAGAATTCGCAATAAAATGCTTGGAGG | Cloning | This study |
| UP-R_vwb_overlap | aagtaattatatttagtatCTGTAAATTCTCCTTAATTTAAACG | Cloning | This study |
| DN-F_vwb_overlap | aaattaaggagaatttacAGATACTAAATATAATTACTTTGCTTC | Cloning | This study |
| DN-R_vwb_NotI | ATCGCGGCCGCTCACATCGCTTAACAATTAACG | Cloning | This study |
| UP_FS600vwb | GCTACAAGCAGCTTAGATCC | Sequencing | This study |
| UP_RS200vwb | TTCTGACTTGTCGATAAATG | Sequencing | This study |
| UP_RS700vwb | GATTCCAACTCTTGATAAC | Sequencing | This study |
| vwb1100DOWN_R | ATTCCCACTCAATGAAATAAG | Cloning | This study |
| vwb1100UP_F | TGTACGATTCTGTGGAATTG | Cloning | This study |
| vwb_50R | ATCGCTATTCCAAAGTTGTG | Cloning | This study |
| vwb_50F | ATACTCTAGGCGAAGCTC | Cloning | This study |
| DN_FS300vwb | ATGGATTGTATTGGCATAAG | Sequencing | This study |
| DN_FS800vwb | GAGGCATCTTCCTTGTCATC | Sequencing | This study |
| DN_RS400vwb | TATTATGAAAGCCCAGTGAC | Sequencing | This study |
| UP_F600scpA | CACTTTACTTCTCCTATTG | Sequencing | This study |
| UP_R200scpA | TAAACTGTGAAGAGTAGAG | Sequencing | This study |
| UP_R700scpA | ATGCCATACCTATGCAATTC | Sequencing | This study |
| UP_R_scpA_overlap | TAAATCTAGATAGTCTTACTTTTCATATAAAAACTCCTTTC | Cloning | This study |
| UP_F_scpA-EcoRI | GAGAATTCGTTTTGACGAATTTTTTGTACG | Cloning | This study |
| scpA1100UP_F | CCATAATGTACCCACTTACG | Cloning | This study |
| DN_F300scpA | AACCTTTCCTCTTCAAATAG | Sequencing | This study |
| DN_F800scpA | GACATAACCAAACCTAAAC | Sequencing | This study |
| DN_F_scpA_overlap | AGGAGTTTTTATATGAAAAGTAAGACTATCTAGATTTATTTAAG | Cloning | This study |
| DN_R400scpA | AAGTATCACATCAAGAAATC | Sequencing | This study |
| DN_R_scpA_NotI | TAGCGGCCGCCCTTATGTCATTCGTAACAG | Cloning | This study |
| scpA1100DOWN_R | GGTTAGTTTGAAGCAGTTTG | Cloning | This study |
| scpA_50R | TCTCAGCGTTTGCAATAGGG | Cloning | This study |
| scpA_50F | ATGAACACAGAAGCTTTAGG | Cloning | This study |
| pKOR1 rev | CACACAGGAAACAGCTATGACATAGTC | Sequencing | [6] |
| pKOR1F outside | CAGCTCCAGATCCATATCCTTC | Sequencing | [6] |
| PLASMID |  |  |  |
| pKOR1 | *E. coli* (AmpR) - *S. aureus* (ChmR) | Shuttle vector for allelic replacement in staphylococci. | [6] |
| pKOR1Δ*vwb* | Construction deletion of *vwbp* for *S. aureus* S0462 |  | This study |
| pKOR1Δ*scpA* | Construction deletion of *scpA* for *S. aureus* S0462 |  | This study |
| STRAINS |  |  |  |
| *S. aureus* S0462 | Pig carrier isolate |  |  |
| *S. aureus* S0462 Δ*vwb* | *vwbp* deletion |  | This study |
| *S. aureus* S0462 Δ*scpA* | *scpA* deletion |  | This study |
| *E. coli* DC10B | *dcm* deficient *E. coli* DH10B, | staphylococcal cloning host allowing  transformation of plasmids directly into *S. aureus* | [7] |
